# Role of Endoplasmic Reticulum Stress, Autophagy, and Inflammation in Cardiovascular Disease

**DOI:** 10.3389/fcvm.2017.00029

**Published:** 2017-05-12

**Authors:** Cheng Zhang, Taha Wasim Syed, Renjing Liu, Jun Yu

**Affiliations:** ^1^Center for Metabolic Disease Research, Department of Physiology, Lewis Katz School of Medicine, Temple University, Philadelphia, PA, USA; ^2^Agnes Ginges Laboratory for Diseases of the Aorta, Centenary Institute, University of Sydney, Camperdown, NSW, Australia; ^3^Sydney Medical School, University of Sydney, Sydney, NSW, Australia

**Keywords:** atherosclerosis, cardiovascular diseases, inflammation, autophagy, endoplasmic reticulum stress

## Abstract

Cardiovascular diseases are a class of heart or blood vessels diseases, which are now considered to be the leading cause of death globally. A number of recent studies have reported key roles for inflammation in the progression of diseased vessels and systematic heart failure. In particular, endoplasmic reticulum (ER) stress, which is mechanistically implicated in inflammation and autophagy, has now been associated with pathophysiological states in the cardiovascular system. Autophagy has also been identified as an important process in the progression of multiple cardiovascular diseases such as in atherosclerosis plaque progression and ischemia and/or reperfusion. In light of the above, it has been proposed that a link between inflammation, autophagy, and ER stress may exist that contribute to diseases of the heart and its supporting vessels. This review highlights current knowledge on the cross talk between these three biological processes, and the potential of targeting this pathway as a therapeutic approach for cardiovascular disorders and its related diseases.

## Introduction

Cardiovascular diseases are the leading cause of mortality in the industrialized world (1) and have become an even greater concern given the aging population. It includes any heart condition that negatively implicates the blood vessels, internal structure or the morphology of heart, essentially contributing to the obstruction of continuous blood supply and nutrients to the heart, and therefore the entire body. Anecdotal evidences strongly suggest that a wide array of inflammatory processes participate in cardiovascular injury as a result of ischemia and/or reperfusion, thrombosis, and infection (2–6). For example, atherosclerosis, a cardiovascular disease stemming from a myriad of different causes and influences including genetic and environmental, has now been identified as a disorder involving chronic inflammation, where inflammation plays a key role in all stages of atherosclerosis from its initiation to its progression and, ultimately to its eventual endpoint in the form of plaque rupture with the detrimental manifestation of atherothrombosis, leading to stroke and ultimately death (7). Despite significant advances in cardiovascular research over the past few years, much work remains to be done to reveal novel targets of therapeutic intervention.

Endoplasmic reticulum (ER) is a membrane-like cellular organelle, continuous with the outer nuclear membrane (8). It forms an interconnected network of space and is primarily responsible for membrane protein translation, posttranscriptional modification of proteins, and upkeep of cellular calcium (Ca^2+^) homeostasis and lipid biosynthesis. ER is now acknowledged as an extremely vital organelle that can poise cells for survival or death based on the cellular stress factors present. In essence, ER is extremely responsive to stressors that exhaust cellular energy or Ca_2+_ levels and that modify the ER luminal redox state. Physiological and pathological insults, such as manifestation of reactive oxygen species (ROS), ischemia/reperfusion, perturbations in Ca^2+^ homeostasis, and release of inflammatory cytokines and toxins, can all lead to a buildup of defective misfolded and unfolded proteins in the ER lumen, a condition referred to as ER stress (9). Essentially, for efficient cardiomyocytes contractile function, protein quality control is indispensable, and any perturbations in protein homeostasis can significantly impact the general cellular function and contractility of the myocytes, resulting in cellular dysfunction and cell death. Since autophagy, a conserved degradation pathway, can be mechanistically triggered by ER stress and inflammation and is known to be involved in atherogenesis, it is also been thought as an important player in the pathogenesis of cardiovascular diseases (10). A deeper understanding of the link between ER stress, inflammation, and autophagy, in relation to cardiovascular diseases, would provide potential new strategies for heart disease prevention and management.

## Inflammation and ER Stress: The Systematic Interplay Between the Two in Pathogenesis of Cardiovascular Diseases

Inflammation, particularly chronic inflammation, has been established as a central underlying driver of many diseases including cardiovascular diseases (3). In terms of atherosclerosis, numerous factors that promote vascular injury include hypertension, smoking, lipid peroxidation, and even infection have been shown to initiate the complex inflammatory cascade, the result of which is a vulnerable plaque, prone to rupture and thrombosis (11, 12), which then leads to myocardial infarction and stroke.

**Figure 1:**
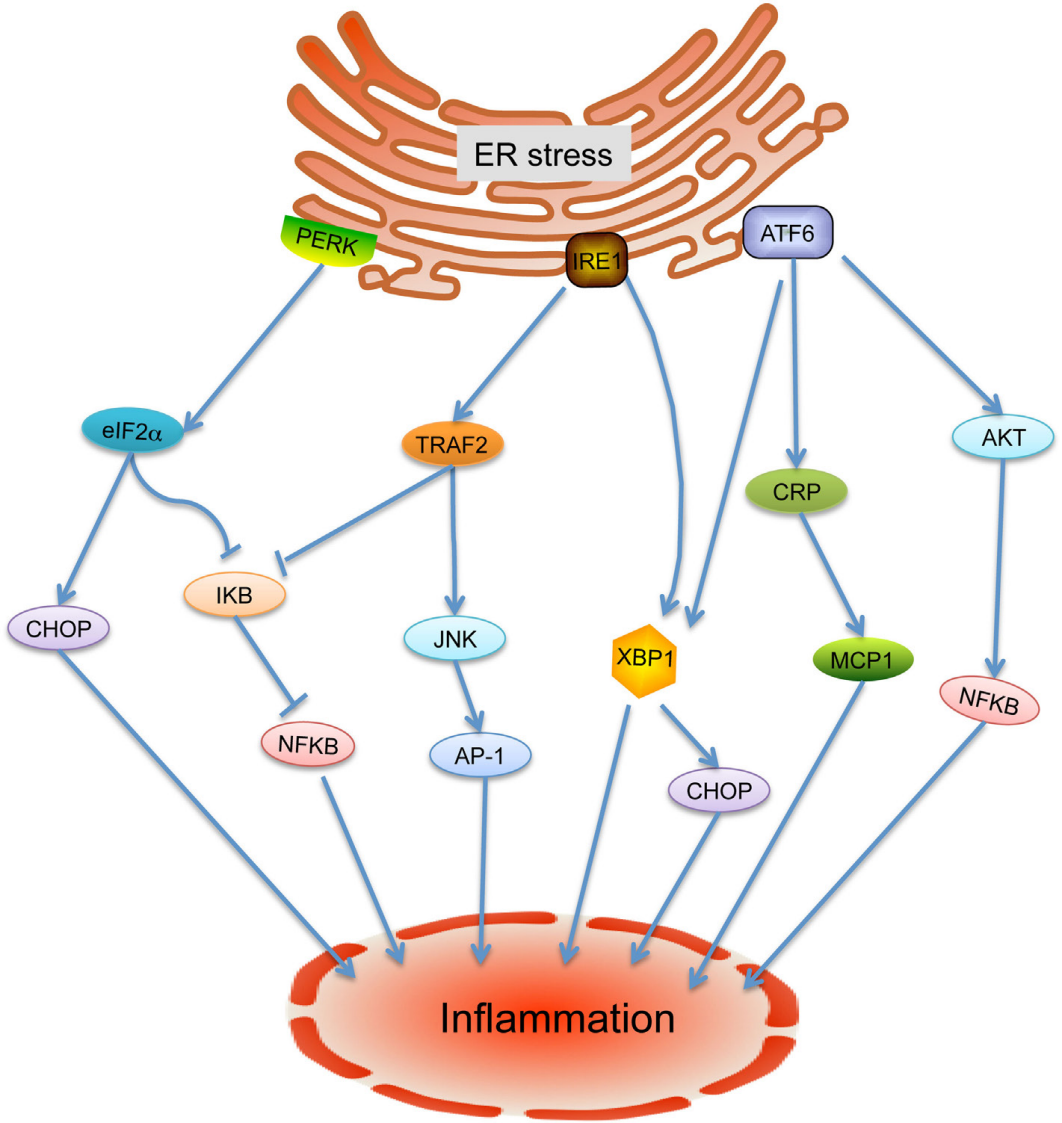
**Endoplasmic reticulum (ER) stress-mediated inflammation**. Under ER stress condition, the unfolded protein response is activated and leads to release of inositol requiring enzyme 1 (IRE-1), PKR-like eukaryotic initiation factor 2a kinase (PERK), and activating transcription factor-6 (ATF6). IRE-1 recruits tumor necrosis factor-α-receptor-associated factor 2 (TRAF2) and causes activation of downstream kinases signaling including Jun amino-terminal kinases (JNK) and nuclear transcription factor-κB (NF-κB), inducing the production of inflammatory cytokines. In addition, the activated IRE-1 also cleaves mRNA encoding X-box binding protein-1 (XBP1) to generate mRNA encoding its active form, causing transcription of inflammation associated gene expressions. Cleavage of ATF6 leads to the activation of XBP1, C-reactive protein (CRP), and Akt to induce the inflammatory response. PERK phosphorylates eIF2a, which leads to the aviation of NF-κB and CHOP to further enhance the expression of inflammatory gene.

Emerging evidence indicates that ER stress is implicated in inflammatory processes and plays rather fundamental roles in the onset and progression of cardiovascular diseases (9). In response to ER stress under challenging cellular conditions, unfolded protein response (UPR) is activated under the mediation of three ER membrane-associated proteins: inositol requiring enzyme 1 (IRE-1), activating transcription factor-6 (ATF6), and PKR-like eukaryotic initiation factor 2a kinase (PERK) (9) (Figure 1). Considerable studies have highlighted that inflammation within the cardiovascular system is involved in ER stress and UPR through a variety of regulators such as nuclear transcription factor-κB (NF-κB), activator protein-1, Jun amino-terminal kinases (JNK), spliced X-box binding protein-1 (XBP1), and ROS (13–16). Under active UPR, the activation of IRE-1 leads its recruitment of tumor necrosis factor-α-receptor-associated factor 2, which in turn interacts with JNK and IκB kinase that then leads to the activation of several downstream inflammatory signals (4, 14, 17, 18). Through autophosphorylation, the intrinsic ribonuclease activity of IRE-1 results in the splice and activation of XBP1, resulting in the production of inflammatory cytokines by enhancing TLR signaling (4). ATF6 has been identified as a synergic mediator between ER stress signaling and pro-inflammatory signaling pathway. In response to ER stress, cleavage of ATF6 leads to transcriptional activation of inflammation related proteins such as C-reactive protein, which can contribute to inflammation by promoting the expression of monocyte chemoattractant protein-1 (MCP-1) receptor (4, 5, 19). In addition, the ATF6 pathway also activates NF-κB through Akt kinase phosphorylation (20). In response to stresses, PERK-eIF2α-mediated translation attenuation of IκB, the inhibitor of NF-κB, results in NF-κB release and activation. Activated NF-κB translocates to the nucleus and then triggers a variety of inflammatory cytokine expressions (21). In addition, the transcription factor CHOP, which activates IL-1β signaling, can be switched on by all three ER stress sensors (3). More importantly, inflammatory factors also have been found to trigger ER stress and UPR activation, suggesting mutual regulation of these two pathways (4).

Histological findings in the failing heart have shown morphological changes in the ER, which indicates that ER overload is implicated in the failing heart (9, 10). Recent evidence suggests that unfavorable cellular conditions such as hypoxia, oxidative stress, and enhanced protein synthesis in diseased hearts could all possibly augment ER stress (22). In humans with heart failure, cardiac ER stress has been demonstrated by the presence of spliced XBP1 and induction of GRP78, ATF4, and CHOP gene expression. Activated IRE-1α splices XBP1 mRNA (23). Spliced form of XBP1 is a basic-region leucine zipper transcription factor that can initiate the UPR in an efficient manner by upregulating genes liable for restoring normal ER function. Quan et al. reported that compared to control, murine hearts subjected to pressure overload and isoproterenol showed adverse cardiac remodeling and a marked increase in myocardial XBP1s protein, with the manifestation of hypertrophy and heart failure (24). In another study, Minamino et al. utilized a model of pressure overload caused by transverse aortic constriction to study the changes in activation of UPR and ER stress-induced proapoptotic signaling in mice (22). Interestingly, they observed upregulation of UPR activation in both hearts exhibiting cardiac hypertrophy and heart failure, whereas stimulation of CHOP associated with ER stress-mediated apoptosis, but not JNK or caspase-12, was found solely in failing hearts (22). Hamada et al. found that ER stress-associated aberrant ER quality control is crucial to the progression of dilated cardiomyopathy in Lys-Asp-Glu-Leu (KDEL) receptor mutant mice (25). The KDEL receptor is a seven-transmembrane-domain repossession protein receptor that plays a significant role in ER quality control, by retro-transporting ER molecular chaperone proteins, components of quality control apparatus, and misfolded proteins, back to the ER, in the early secretory pathway. Hamada and colleagues observed that cardiomyocytes from mutant mice under treatment with tunicamycin were sensitive to ER stress and manifested impairment in the L-type Ca^2+^ current (*I*_Ca,L_) (25). Detailed structural analyses further revealed expanded sarcoplasmic reticulum and ubiquitinated protein aggregates. Among other things, in the mutant hearts, they also noticed overexpression of CHOP and increased cellular apoptosis. Collectively, the data suggest that ER stress and UPR activation is persistent in hearts subjected to pressure burden (9).

Atherosclerosis is a major cardiovascular disorder that is characterized by plaque buildup inside the arteries. Although the atherosclerotic process is not fully understood, it is well established that ER stress is involved in the disease (3, 22, 26). Activations of UPR are observed in atherosclerotic plaques at all stage of atherogenesis, especially in macrophage- and SMC-derived foam cells (9). Moreover, stressors including oxysterols, oxidative stress, saturated fatty acids, and high levels of intracellular cholesterol in advanced lesions can also result in prolonged activation of the UPR (3). Mechanistically, there are a number of different ways in which aberrant ER function may impact the progression of atherosclerosis. For example, UPR can affect lipid metabolism since several vital lipogenic pathways are located in the ER (27), and XBP1 has been shown to participate in ER phosphatidylcholine synthesis (9). Under ER stress conditions, the lipogenesis and hepatic lipid accumulation were negatively activated (28). Recent evidence supports the view that effective rescue of ER stress by means of molecular or chemical agents/chaperones can ardently avert and repress hepatic lipid accretion and assist in maintaining lipoprotein secretion, inducing other protective effects against the pathogenesis of wide variety of cardiovascular diseases (9). On the contrary, regulating membrane lipid metabolism by suppressing biosynthesis of phospholipids or *via* amplifying the phospholipid hydrolyzing function of phospholipase aggravates perturbation of ER homeostasis and modulates sphingolipid levels, which can in turn negatively affect proper ER function (29, 30). A growing body of evidence suggests that perturbations in cardiac muscle tissue or surrounding vessels can trigger an inflammatory response through the recruitment of inflammatory cells (such as macrophages and neutrophils) to the site of damage, which subsequently can lead to the production of pro-inflammatory cytokines (IL1 and IL6) along with the formation of ROS. The release of these ROS along with inflammatory cytokines could then initiate the ER stress at the site of plaque (7). Due to the inherent nature and function of inflammatory cells, these cell types traffic vast amounts of protein-cargo through the ER, and thus are extremely susceptible to prolonged alterations in ER homeostasis, and any overwhelming changes in the ER thereby can induce ER stress-based inflammation and macrophage death leading to the disruption and rupture of vascular plaques (9). The current model suggests that changes in lipid status and cellular exposure that constitutes physiological stresses and cardiac burden can prompt ER in its function to aptly convey this stress-related information to signaling pathways linked to inflammation and cell death, in a variety of cell types, including macrophages and neutrophils, eventually promoting pathogenesis and progression of ruptured atherosclerotic plaques.

Ischemic heart disease (*aka*, coronary artery disease) is another leading cause of death and is caused by reduced blood supply to the heart. The infiltration of monocytes/macrophages is thought to play a critical role in the pathophysiology of this disease. Therefore, ischemic heart disease is widely conceived to be an inflammatory disease (31, 32). Moreover, during the development of ischemic heart disease, the onset of ER stress results in the degeneration of cardiac myocytes leading to cell death (33). It has been reported that cardiac-specific expression of MCP-1 in mice causes a cluster of ER stress-related gene being transcriptionally activated in the heart, which as a result then leads to the development of ischemic heart disease (32). Thus, the ER stress pathway is also implicated in the pathogenesis of ischemic heart disease, most likely through induction of apoptosis or inflammation.

Under physiological conditions, ER stress-initiated inflammation aims to primarily reduce any tissue damage and aid in tissue repair. However, either under overwhelming or excessively stressful cellular conditions, ER stress can negatively promotes the progression and pathogenesis of cardiovascular diseases.

## Role of Autophagy in Cardiovascular Diseases

Autophagy is a highly conserved lysosomal catabolic pathway responsible for the degradation of intracellular proteins and disposal of damaged organelles. Active autophagic activity has been reported in all vascular cell types including smooth muscle cells, endothelial cells, macrophage, cardiomyocytes, cardiac fibroblasts, and myofibroblasts that make up the circulatory system (34). Due to the critical role of autophagic processes in the maintenance of cell health, it is not surprising that perturbations of autophagy are responsible for the cardiovascular-related physiology and pathology. Autophagy plays dual roles in the cardiovascular system where *physiological* autophagy is protective and required to maintain normal cardiovascular function, while *pathologic* autophagy is involved in the manifestation of cardiovascular disease (35, 36). Hence, autophagy may emerge as a therapeutic target of clinical relevance.

In advanced atherosclerosis plaques, autophagy-like ultrastructural features and expression of autophagy marker LC3-II were found (10). Furthermore, the factors that induce atherosclerotic plaques such as oxidized lipids, inflammation, and metabolic stress conditions can also stimulate the autophagy (37). This suggests that autophagy is a major component in the progression of atherosclerosis. Although the function of autophagy in atherosclerosis plaque formation and stability is poorly understood, it is worthy to note that autophagy in atherosclerotic plaques could be either beneficial or detrimental (10). Emerging data reveal protective roles of autophagy in the regulation of atherosclerotic plaque development where basic/physiological autophagy is thought to be a survival mechanism that helps plaque cell against stress conditions by eliminating damaged intracellular material (38). In progression of atherosclerosis, defective autophagy was shown to enhance apoptosis (39). Treatment of vascular smooth muscle cells with 7-ketocholesterol, an oxysterol present in plaques, attenuated statin induced cell death by activating autophagy (34). Similarly, autophagy was induced in endothelial cells in the presence of oxLDL or lipid peroxidation products to protect against cell injury (40). Moreover, verapamil, a Ca_2+_ channel blocker that is used to relax the muscles of heart and blood vessels, has been shown to be beneficial in controlling vascular injury-induced neointimal formation through its autophagy related antiproliferative effect in smooth muscle (41). Most importantly, macrophage-specific ATG5 null mice exhibited enlarged plaques by inducing inflammasome activation and an increase in cholesterol crystal formation, suggesting an essential role for basal levels of autophagy in atheroprotection (39, 42). In addition, autophagy deficient macrophages have shown an attenuated capacity to regulate cellular cholesterol. Separately, deletion of autophagy inhibitor Wip1 can positively modulate lipid metabolism and reduce the atherosclerotic plaques (43). These findings indicate that autophagy might have a protective role in the progression of atherosclerosis; however, excessive autophagic activity can also exhibit detrimental effect by destroying major cytosolic organelles and proteins (37). It has also been reported that overactive autophagic activation may cause smooth muscle cells and endothelial cells death finally leading to thinner fibrous cap, plaque destabilization, thrombosis, and acute myocardial infarction (37, 43, 44). Furthermore, autophagic cell death has been found to lead to immunogenic response and release of inflammatory factors (43).

Abundant reports have shown that a number of autophagosomes increase in the heart during ischemia/reperfusion injury (45–49). In the case of myocardial ischemia/reperfusion injury, the nutrient and oxygen supplies to the heart are limited. Under this starvation state, it is thought that autophagy is triggered (49) and serves as a homeostatic mechanism to protect against the consequences of further ischemia (50). For instance, following initiation of autophagy, free fatty acids and amino acids are released and recycled to generate ATP, to subsequently compensate for the energy deficiency under ischemia. However, the autophagy inhibitor, 3-MA, can attenuate ATP generation and can lead to cardiac cell death in response to nutrition deprivation (49). In addition, many compounds that have cardioprotective function also induce autophagy such as rapamycin and statins (51–53). As in atherosclerosis, autophagy is also a double-edged sword in ischemia/reperfusion injury. While autophagy serves as a homeostatic mechanism to provide energy to the injured heart, uncontrolled excessive autophagic activity may lead to cellular dysfunction or death. For example, decreased expression of autophagy inhibitor Bcl-2 or overexpression of beclin1 may contribute to apoptotic cell death and accentuated pathological remodeling induced by severe pressure stress (49, 54).

Taken together, the function of autophagy in cardiovascular and other diseases is not as simple as beneficial or detrimental. The context-dependent role of autophagy in disease development has to be considered.

It should also be noted that some autophagy proteins may exert autophagy-independent functions, such as apoptosis. For example, ATG5, which is required for the formation of the autophagosome, is also involved in apoptosis regulation. ATG5 fragment that is cleaved by calpain has proapoptotic properties (55). In addition, Bcl-2 is a dual regulator of autophagy and apoptosis (56). Since apoptosis also play important roles in the heart disease, cross talk must exist between autophagy and apoptosis during the pathologic development of cardiovascular disease. Moreover, it has been shown that depletion of different Atg genes will lead to distinct cardiac defects in mice, further suggesting that these genes may exert autophagy-independent functions (57–59).

## ER Stress, Inflammation, and Autophagy: Implications of Aberrant Cellular Cross Talk

Autophagy is a highly regulated intracellular degradation system engaged in the turnover of intracellular proteins and damaged organelles (60–62). Although ER stress and autophagy can function independently, mounting evidence indicates that autophagy is interrelated to the ER at many levels. Since ER has often been observed in close vicinity of the isolation membrane, it has been considered as one of the major sources of the autophagosome or the platform for autophagosome formation (63). The fragments of ER membrane are found to transport to the autophagosomes under starvation conditions (64). Molecules in the autophagy cascade from ULK1 to PI3P effectors, which are responsible for autophagosome nucleation are localized to the ER, suggesting that ER is likely a candidate for the membrane source and the scaffold for autophagosome formation (63, 65–67). As the two systems are dynamically interconnected, altering the functions of one can influence the other. As mentioned above, ER stress is an important cause of advanced lesional macrophage apoptosis resulting in atherosclerotic plaque necrosis. Interestingly, the ER stress is also a trigger of autophagy (68). Under ER stress, the autophagy is induced through the IRE-1–JNK/p38 pathway and the ATF4 pathway (3) in mammalian cells. However, pre-activation of autophagy by ischemic preconditioning can activate defense mechanisms and reduce excessive ER stress (69).

Recent developments reveal a crucial role for the autophagy pathway in inflammation. A major effect is on the level of inflammasome. Increased production of IL-1β and IL-18 was found in LPS stimulated macrophages isolated from the Atg16L1 or Atg7 deficient mouse (61, 70), where activated autophagy effectively cleared the danger signals such as mitochondrial ROS and DNA that promote the inflammasome (62). Another important effect of autophagy on inflammatory system is the regulation of inflammatory transcriptional responses. In autophagy defective cells, the pro-inflammatory transcription factor NF-κB was found to be activated (62). Specifically mice with macrophage-specific knockout of ATG5 had increased plaque formation, revealing an important role for basal levels of autophagy in atheroprotection. Further investigation showed that defective autophagy in ATG5 knockout macrophage is linked to proatherogenic inflammasome activation. The basal level autophagy serves an important function for the cardiomyocytes and this is supported by a study suggesting that heart-specific depletion of the autophagy protein Atg5 causes cardiomyopathy in mice (71). These studies suggest that ER stress, autophagy, and inflammation may coordinately play a function in the progression of heart clinical events.

Though growing data suggest that cross talk exist between ER stress, autophagy, and inflammation, the molecular interplay among the three pathways in cardiovascular disease is yet to be further defined (42). The reticulon (Rtn) family protein is a large and diverse group of membrane-associated proteins that are primarily localized to the ER (72, 73). In mammalian cells, there are four Rtn genes: Rtn-1, -2, -3, and -4, with each gene encoding multiple isoforms (72, 74). It has been reported that CHOP, ATF6, and other ER stress-specific transcription factors were sufficient to upregulate the expression of RTN3 suggesting that the protein may participate in ER stress regulation (75). In addition, inhibition of expression of RTN3 enhances the induction of autophagy resulting from cyPrP aggregates, which then can lead to ER stress, thus revealing a relationship between this protein and ER stress-related autophagy (76). The Rtn-4 family consists of three splice variants of a common gene called Rtn-4A, Rtn-4B, and Rtn-4C (73). Rtn-4B, also known as Nogo-B, is ubiquitously expressed in many cell types including monocytes/macrophages (73). Previously, we have reported the role of Nogo-B in controlling apoptosis and inflammation regulation (73). Nogo-B essentially changed the localization of antiapoptotic proteins Bcl-2 and Bcl-XL to the ER and reduced their antiapoptotic activity in D98/AH2 cells through as yet unknown mechanisms (77). Moreover, in pulmonary arterial hypertension, Nogo-B induction disrupts the ER-mitochondria unit by increasing the distance between the ER and mitochondria and suppresses apoptosis (78). Together, these findings indicate Nogo-B to be a very important ER protein that can regulate ER morphology, but its role in autophagy regulation has not been investigated.

## Conclusion

**Figure 2:**
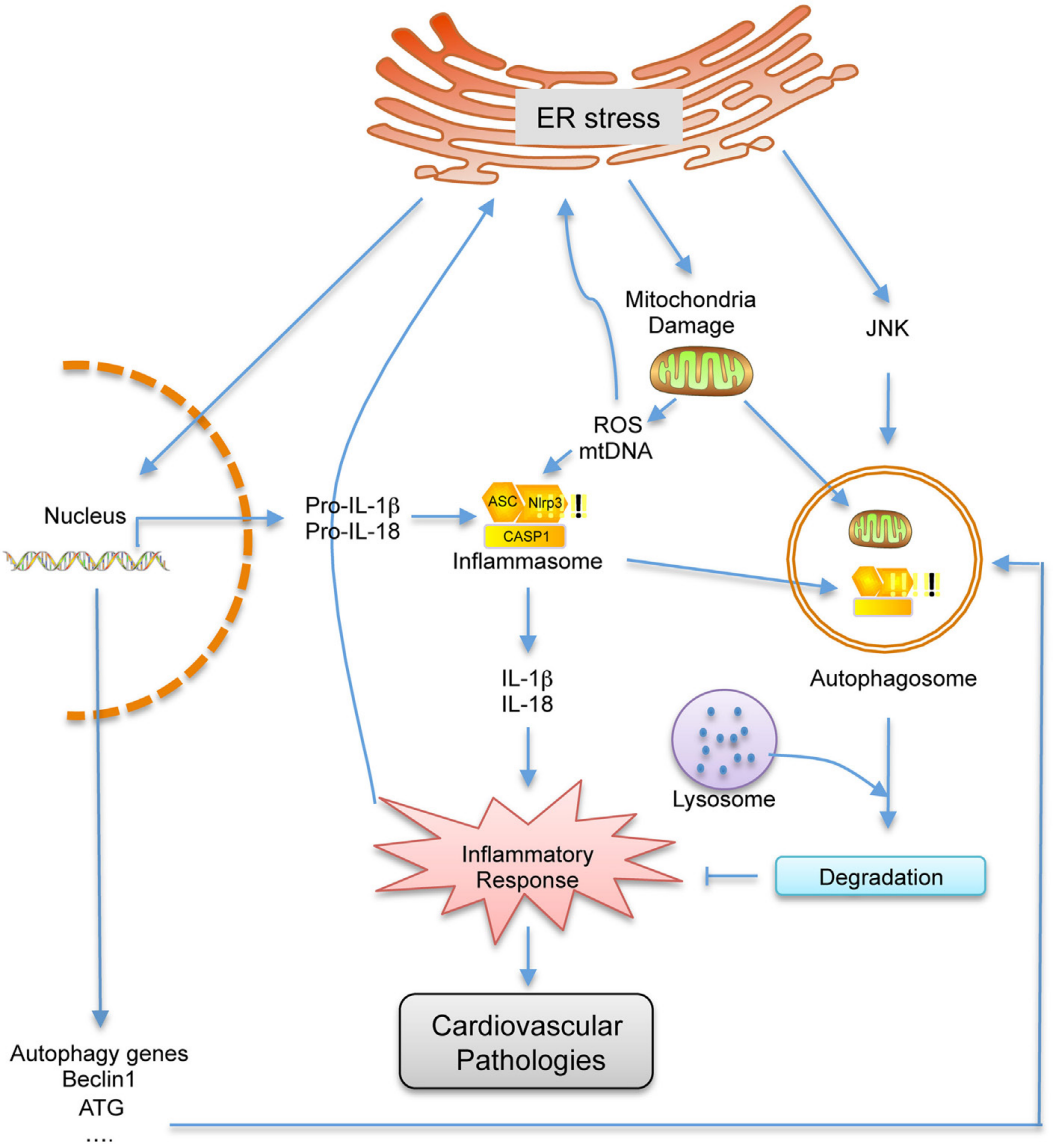
**Schematic representation of the cross talk between endoplasmic reticulum (ER) stress, autophagy and inflammation**. Under ER stress conditions, the inflammatory response, which can contribute to cardiovascular disease, is activated by the damaged mitochondria and elevated pro-inflammatory gene expressions. As a feedback, inflammatory response can further enhance ER stress. However, autophagy, which is triggered by ER stress, can inhibit inflammatory response by degrading damaged mitochondrial and inflammation related proteins, which can also further inhibit ER stress. Therefore, ER stress, autophagy, and inflammation are closely related, and one can influence the other.

The ER stress-related inflammation and autophagy play a key role in the pathogenesis of various diseases, including cardiovascular disease. Although our understanding of the roles of these three processes in the progression of cardiovascular disease has progressed in recent years, the detailed molecular mechanisms and cellular pathways at play is still in its infancy and important questions remain unanswered. Here, we have reviewed published studies that indicate a link between these three biological processes at the molecular and physiological levels and highlight the importance of the interplay between them in the progression of cardiovascular disease. In summary, ER stress, inflammation, and autophagy are tightly integrated and each one can influence the other (Figure 2). As a result, it can be hypothesized that these fundamental cellular pathways are maintained in a delicate balance state to respond to specific environmental fluctuations. Once the balance is disrupted, it can lead to a variety of pathologies, including many important cardiovascular diseases such as atherosclerosis. Therefore, deeper understanding of the interconnection between these three physiological processes especially under pathological conditions will be of great importance and may shed light in developing new strategies in treating cardiovascular diseases.

## Author Contributions

CZ and JY designed the study. CZ, TS, and JY wrote the manuscript. RL and JY edited the manuscript.

## Conflict of Interest Statement

The authors declare that the research was conducted in the absence of any commercial or financial relationships that could be construed as a potential conflict of interest.
